# Infection control measures in nosocomial MRSA outbreaks—Results of a systematic analysis

**DOI:** 10.1371/journal.pone.0249837

**Published:** 2021-04-07

**Authors:** Béke Pannewick, Claas Baier, Frank Schwab, Ralf-Peter Vonberg

**Affiliations:** 1 Institute for Medical Microbiology and Hospital Epidemiology, Hannover Medical School, Hannover, Germany; 2 Institute for Hygiene and Environmental Health, Charité –University Medicine Berlin, Berlin, Germany; The Rockefeller University, UNITED STATES

## Abstract

There is a lack of data on factors that contribute to the implementation of hygiene measures during nosocomial outbreaks (NO) caused by Methicillin-resistant *Staphylococcus aureus* (MRSA). Therefore, we first conducted a systematic literature analysis to identify MRSA outbreak reports. The expenditure for infection control in each outbreak was then evaluated by a weighted cumulative hygiene score (WCHS). Effects of factors on this score were determined by multivariable linear regression analysis. 104 NO got included, mostly from neonatology (n = 32), surgery (n = 27), internal medicine and burn units (n = 10 each), including 4,361 patients (thereof 657 infections and 73 deaths) and 279 employees. The outbreak sources remained unknown in 10 NO and were not reported from further 61 NO. The national MRSA prevalence did not correlate with the WCHS (p = .714). There were significant WCHS differences for internal medicine (p = 0.014), burn units (p<0.01), for Japanese NO (p<0.01), and NO with an unknown source (p<0.01). In sum, management of a NO due to MRSA does not depend on the local MRSA burden. However, differences of MRSA management among medical departments do exist. Strict adherence to the Outbreak Reports and Intervention Studies Of Nosocomial infection (ORION) statement is highly recommended for. The WCHS may also serve as a useful tool to quantify infection control effort and could therefore be used for further investigations.

## Introduction

Methicillin-resistant *Staphylococcus aureus* (MRSA) is a well-known pathogen causing large numbers of sporadic nosocomial infections each year worldwide [1]. MRSA is also known as one of the most important causes of nosocomial outbreaks (NO) with significant morbidity and mortality. That is why numerous national and international infection control guidelines are provided to assist infection control staff when confronted with such an event [2–4]. Some of these recommended measures may get implemented or enforced quite easily, such as the use of protective clothing and temporary improvement of hand hygiene compliance. However, other measures may be much more difficult, demanding and/or expensive, for example screening of staff, improvement of the staff-to-patient ratio, or the total closure of a unit or ward for new admissions.

The burden of nosocomial MRSA varies considerably between different countries. For example, within Europe, the MRSA prevalence tends to be rather low for decades in the Netherlands and the entire Scandinavian area. In contrast, MRSA prevalence rates are moderate (e.g., Spain, France, and Germany) or even fairly high (e.g., Italy, Greece, or Portugal) in other countries [5].

Countries with extraordinary low MRSA rates often perform a so-called “search and destroy” strategy in the endemic setting. Patients being at increased risk for MRSA positivity are primarily placed in a single room until a (hopefully negative) screening result is available minimizing the risk for MRSA transmission [6–8]. Unfortunately, until now only little is known about potential discrepancies of national efforts to combat MRSA transmission in an epidemic situation (outbreak). This systematic analysis of the medical literature closes this gap of information as it provides a detailed insight into the management of NO due to MRSA particularly with regard to national MRSA prevalence rates and to other characteristics of such outbreaks.

## Materials and methods

### Databases for outbreak reports

The Outbreak Database (www.outbreak-database.com) served as the primary source for MRSA NO reports, as it represents the world’s largest collection of all kinds of NO with more than 3,600 outbreak reports [9,10]. The collection of data was then completed by a supplementary literature search of PubMed (http://www.pubmed.gov) and Embase (https://www.embase.com) with a search strategy applied as follows: (nosocomial) AND (outbreak OR epidemic) AND (MRSA OR methicillin). Finally, the reference lists of all articles were then checked for any further outbreak descriptions not yet included in the analysis. The first searches took place on February 20^th^ 2019 and were last updated on April 13^th^ 2020.

### Inclusion criteria for outbreak reports

Only descriptions of a solitaire NO of MRSA were included. For comprehension reasons, articles had to be published in English, French, or German language. Focus of the analysis at hand was the US, Canada, entire Europe, and Japan as the number of NO reports from other geographical areas was too low for further systematic evaluation. Only articles published in or after the year 2000 were included in order to dismiss inappropriate historical conditions and providing timely data only. Summed-up results from other reviews got excluded in order to avoid data selection bias.

### Data collection from outbreak reports

#### Setting

Data on the year of the NO, the country, the type of hospital (children´s hospital, general hospital, teaching hospital, long time care facility, or university hospital) and unit (intensive care vs. peripheral ward), and the medical discipline(s) got determined.

#### Infections

The numbers of colonized and infected persons including the types of infections and the routes of transmission were extracted.

#### Infection control measures

As mentioned above, some measures require more effort than do others. Therefore, a sum score (weighted cumulative hygiene score; WCHS) for measures clearly mentioned in the NO reports was generated to address this issue adding up to 21 for a maximum score possible (Table 1). The assumed relevance of the infection control measures in this score was based on experience in the field of infection control.

**Table 1:** Weighted cumulative hygiene score (WCHS): Sum score for the assumed relative effort of certain infection control measures.

Measure	Score	Comment on the Rationale for Scoring
Screening of patients	1	• easy to implement • moderately cost only
Screening of personnel	3	• potential legal issue due to interference with personal rights of medical staff
Screening of environment	2	• easy to perform • somewhat expensive as there is no specific part of the environment to look for MRSA
Isolation (single room or cohort)	1	• part of almost every guideline on the care for MRSA positive patients • most probably in place already
Change of antibiotics	1	• there are substances thought to promote MRSA selection such as fluoroquinolones • more often the change of antibiotics will be a consequence rather than a preventive measure
Improvement of hand hygiene	1	• easy to aim for • all equipment needed is in place already anyway
Protective clothing	1	• part of almost every guideline on the care for MRSA positive patients • most probably in place already
Changes in disinfection and/or sterilization procedures	2	• inactivation of staphylococci is rather easy • might be due to wrong concentration of disinfectant • might be due to some technical failure of a device • overall not very likely to occur • most of the times rather easy to fix if noticed
Changes in handling of medical devices	2	• might include an exchange of a contaminated lot • might include the need for repairing • difficult to predict if easy or hard to fix
Training and education of staff	1	• routine measure in all outbreak investigations • no additional cost
Closure of the ward/department	3	• only rarely performed • quite expensive to close an entire unit for admissions of new patients
Improvement of staff-to-patient ratio	3	• expensive measure as the number of staff would need to increase while the reimbursement for the patient care remains unchanged
**Infection control sum score**	**21**	

### Databases for national MRSA prevalence

The weighted cumulative hygiene score was correlated with the corresponding national MRSA prevalence (percentage of MRSA on all *Staphylococcus aureus* isolates) at the beginning of the outbreak. Sources for MRSA prevalence data were the Center For Disease Dynamics, Economics & Policy (CDDEP http://resistancemap.cddep.org; CDC; www.cdc.gov), the European Antimicrobial Resistance Surveillance System (EARSS) provided by the European Centre for Disease Prevention and Control (ECDC; www.ecdc.eu), the Canadian Antimicrobial Resistance Alliance (CARA; www.can-r.com), and the Japan Nosocomial Infections Surveillance (JANIS; https://janis.mhlw.go.jp/english). All of those official databases provide annual data on the proportion of MRSA on the total number of *S*. *aureus* strains in the clinical setting.

### Statistical analysis

We investigated the correlation between the national MRSA prevalence and other NO-related factors to the WHCS. In the descriptive analysis, continuous variables were categorized into categorical variables. Variables were specified as absolute numbers, percentages, median and interquartile range where applicable. Differences were tested by X² test or Wilcoxon rank-sum test. To analyze the WCHS, we calculated univariable logistic regression models for all variables with the outcome WCHS ≥ median. To calculate the effect and independence of factors associated with the WCHS, a multivariable linear regression model was calculated by stepwise backward variable selection. All variables with p<0.25 in the univariable logistic regression model were included in a full model and variable with p-value of type III test ≥0.05 were excluded from the model. Depending on the knowledge of the analyzed factors known before or during/after an outbreak, we calculated two different models. Analyses were exploratory in nature. All analyses were done with SPSS (version 25) and SAS (version 9.4).

## Results

### MRSA prevalence

**Fig 1:**
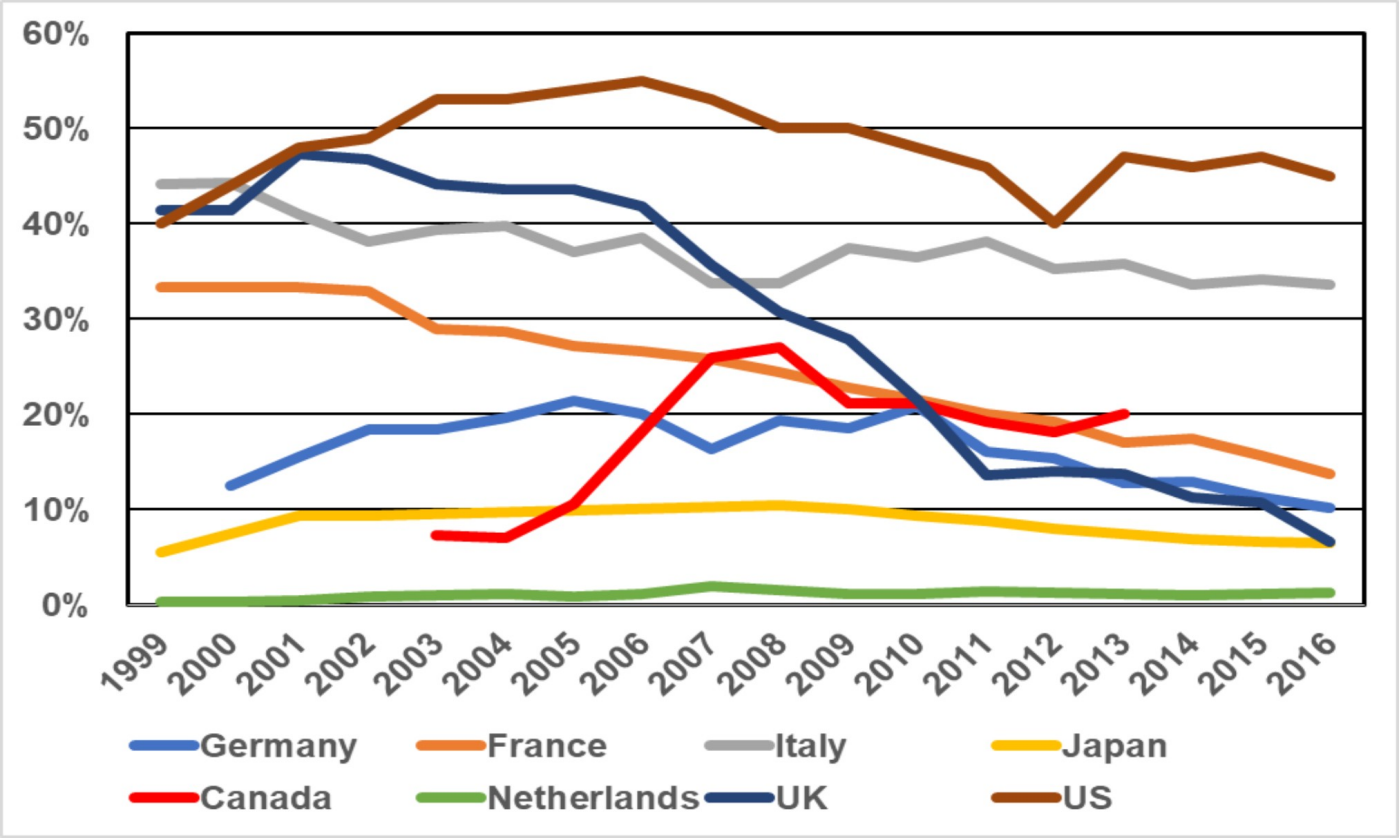
National MRSA prevalence of the eight countries most often affected by nosocomial MRSA outbreaks.

Fig 1 shows the distribution of the national MRSA prevalence (percentage of MRSA on all *Staphylococcus aureus* isolates) over time for the 8 countries most often affected by nosocomial MRSA outbreaks: US, UK, France, Canada, Japan, Italy, the Netherlands and Germany.

### Outbreak settings

A total of 104 outbreak descriptions from 18 different countries were included. A complete reference list of these articles is provided as supplemental material (S1–S3 Appendices). Most outbreaks occurred in the US (n = 21), the UK (n = 15), France (n = 14) as well as Canada and Japan (n = 8 each). The longest duration of an MRSA outbreak was 8.5 years,^11^ while the average length of NO was 7 months (median = 7 months). Among the medical disciplines, neonatology (n = 32), surgery (n = 27), internal medicine, burn units (n = 10 each) and gynecology (n = 5) were most often affected by NO due to MRSA. In addition, intensive care units (ICU) were affected in 40% of all outbreak reports. 35% of the nosocomial MRSA outbreaks were reported from university hospitals.

In 38 of the 104 NO (37%), general MRSA admission screening was carried out; in 12 NO a screening was not performed and in 54 reports no information on this topic were provided. The main route of MRSA transmission explicitly mentioned was contact (n = 25), either direct contact via medical staff (n = 15) or indirect contact via contaminated surfaces in the environment, and medical equipment (n = 6). In four cases the transmission occurred directly from patients. The authors of five NO provided any information on costs. Two reports reported outbreak related costs with an average of € 44,179 per NO [11,12]. The remaining three each provided information on total costs, total loss and cost savings [13–15].

### Involved persons and infections

A total of 4,361 patients and 279 staff members were involved in the outbreaks. In average, 45 patients per outbreak (median = 15; range: 3 to 1,771) were found positive for the outbreak strain. There were 657 documented infections, mainly skin/soft tissue and wound infections (n = 50), endocarditis and blood stream infections (n = 43), pneumonia and other deep respiratory tract infections (n = 20), urinary tract infections (n = 7), and infections of the central nervous system (n = 3). 73 patients deceased due to the course of their MRSA infection.

### Molecular typing

In 89 NO, information on molecular typing procedures was provided. Various genotyping methods were used, most commonly pulsed field gel electrophoresis (PFGE; n = 69), multi locus sequence typing (MLST; n = 19) and other polymerase chain reaction amplification (PCR)-based techniques (n = 17). Phage typing was performed in 8 NO. Other procedures were implemented in 6 cases. 15 NO did not provide information on typing at all. Looking at MLST the predominant MRSA clonal complexes (CC) were CC8 (n = 20), CC22 (n = 10), CC5 (n = 8), and CC30 (n = 4); the predominant clonal lineages were ST8 (n = 13), ST22 (n = 10), ST5 (n = 6), and ST247 (n = 4).

### Infection control measures

**Fig 2:**
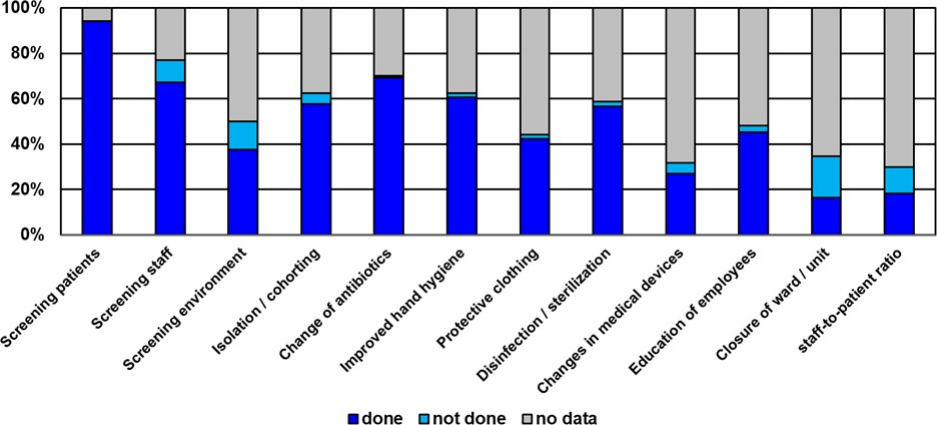
Infection control measures in nosocomial MRSA outbreaks.

Usually a bundle of infection control measures got implemented in the outbreaks included in this study. Fig 2 shows the distribution of these measures. While screening of patients was part of the investigation of most NO, the closure of a ward or an improved staffing was only rarely done. The median weighted cumulative hygiene score determined as described above was 10 (IQR: 6–12).

**Fig 3:**
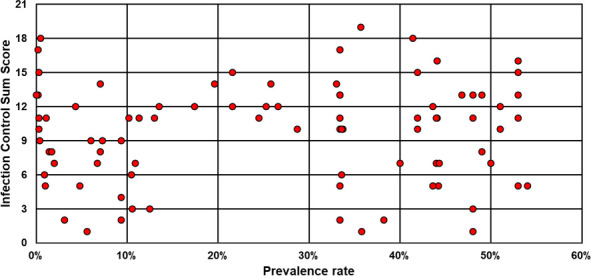
Infection control effort in nosocomial MRSA outbreaks depending upon the national MRSA prevalence rate (Spearman correlation coefficient 0.039; p = 0.714).

As shown in Fig 3, the national MRSA prevalence in the year of the outbreak did not correlate with the efforts in infection control (Spearman correlation coefficient = 0.039; p = 0.714).

Table 2 shows the results of the multivariate linear regression analysis for the endpoint of a high weighted cumulative hygiene score. Additional information on the univariate and multivariate analysis is provided as supplemental material (S1 and S2 Tables).

**Table 2:** Multivariate linear regression analysis related to an infection control score above the median.

Factor	Regression coefficient	p-value	Confidence interval (CI 95%)	Availability on data on infection control
Source was not mentioned	-3.846	0.000	(-5.455; -2.237)	41.4%
Report from Japan	-4.837	0.002	(-7.821; -1.853)	33.3%
Burn unit	3.613	0.009	(0.922; 6.304)	65.8%
Internal medicine	-3.387	0.014	(-6.078; -0.696)	36.7%

Burn units were associated with an elevated score, while infection control efforts were rather scarce in internal medicine, in Japanese hospitals or if the source of the outbreak remained unknown. A separate comparison of the impact on the infection control score, of variables that were already known at the onset of the NO (e.g., country or type of medical department) to variables that became obvious during the course of the NO (e.g., source, route of transmission, or number of patients) is shown in the supplemental material.

## Discussion

Up to now, NO caused by MRSA represent a relevant challenge from an infection control point of view and contribute to a relevant burden of diseases worldwide [1]. When talking of European countries only, there are some 170,000 MRSA infections annually, but there were just 104 NO reports matching our inclusion criteria recorded in international data bases in the recent 20 years [16]^.^ Considering this obvious discrepancy it seems safe assuming that a large number of NO remain out of sight for outbreak research. This may partly be due to a general decreasing interest in both authors and editors, as MRSA has become a rather common pathogen over the last decades. However, MRSA prevalence remains high in many countries as shown in Fig 3 and so does its clinical and economic impact. This should be kept in mind when dealing with future events of MRSA outbreaks as publishing those in detail is herewith highly recommended. In particular it is important to identify the source of the outbreak as this will likely effect the success of infection control (Table 2).

This systematic NO analysis found a dependence of the type of medical specialty on our weighted infection control score. Pasricha et al. showed in a multivariate regression analysis that transferal from an internal medicine ward was an independent risk factor for being missed by MRSA screening [17]. Our data show similar results, as we found a rather low infection control effort in internal medicine units. One may speculate that this infection control reluctance could be attributed to plain resignation caused by an extraordinary high MRSA burden in this medical field. A recent systematic review by the Cochrane Centre aimed to assess the effectiveness of wearing gloves, a gown or a mask when caring for MRSA positive patients. Despite the widely recommended use of protective clothing in guidelines, they failed to find any eligible studies on this topic, either completed or ongoing. This lack of evidence in the endemic setting may diminish infection control compliance in the epidemic setting, too [18]. Furthermore, financial considerations may also play an important role, as universal MRSA screening is economically burdensome in certain hospital settings, although our data is insufficient to prove this assumption [19].

In contrast, burn units were highly associated with MRSA infection control effort including environmental screening and closure of the entire ward. We refer this observation to two reasons: Firstly, we postulate an elevated MRSA awareness because of the severity of illness and increased risk of wound infections after burn injuries, especially due to MRSA and other multi drug resistant pathogens [20,21]. Secondly, it is known that the staff-to-patient ratio is often improved and large space cubicles are used for patient care in such units [22]. These features may facilitate infection control compliance.

Surprisingly, the MRSA prevalence did not influence MRSA infection control effort as mentioned above. However, there was an association between some countries and management of NO caused by MRSA as shown in Table 2. In particular, NO reports from Japan contained significantly less specific information on infection control measures [23–27]. When checking those articles in more detail, we found that they often focused on typing methods rather than providing full information on infection control issues. Whether this is due to general publication bias from the Asian region [28] or to other reasons remains unsettled. Publication bias is also a limitation for the study at hand. Specifically the high proportion of university hospitals and ICUs raise suspicion. On the one hand, the case mix index of patients may be higher in those areas, thus increasing the likelihood for MRSA appearance and familiarity with MRSA surveillance [29], but these facts seems somewhat insufficient to explain their involvement in such a great extent. Also to be considered is a possible bias with regard to the countries of the NO. In this context, more and more publications from countries with corresponding research opportunities and financial resources are being written. Those that lack these resources but may also be affected by MRSA cannot be included. Finally, a large number of the publications did not provide detailed information on infection control measures at all (Fig 2), which is very contradictory to the recommendations for Outbreak Reports and Intervention studies Of Nosocomial infection (ORION) as proposed by Stone et al. [30]. Therefore we would like to encourage authors of further outbreak reports to meticulously adhere to this ORION guideline in order to make all relevant data available for upcoming outbreak research.

## Conclusion

Several needs remain. First, there is the need for proper MRSA infection control management in NO continues regardless of the local or national MRSA burden. This comes with the second need, which is the need for controlled intervention studies addressing MRSA infection control measures to back up corresponding recommendations in guidelines–at least in endemic settings. The third need deals with publishing of NO reports in general. Although most probably available, many reports lack highly important information. The use of a checklist as in the ORION statement would easily close this gap if applied by authors, reviewers, and editors. The option of (partly) anonymous publication may be helpful for authors who fear judicial consequences from reporting.

Finally we would like to recommend the Outbreak Database as a most valuable tool for all people interested in NO, including but not limited to infection control specialists, clinical personnel, and staff in medical training. This database can be extremely helpful for the purpose of infection prevention as well as during an ongoing outbreak investigation.

## Supporting information

S1 Table Descriptive analysis of factors recorded for the MRSA outbreaks and stratifying by the weighted cumulative hygiene score above the median (univariate analysis). (DOCX)

S2 Table Multivariate analysis by linear regression analysis for the weighted cumulative hygiene score. (DOCX)

S1 Appendix List of all MRSA outbreak reports included. (DOCX)

S2 Appendix Raw data of all MRSA outbreak reports included. (XLS)

S3 Appendix PRISMA flow diagram. (DOC)

S4 Appendix PRISMA check list. (DOC)
