# Supplementary material for: Infection control measures in nosocomial MRSA outbreaks—Results of a systematic analysis
Source: PLoS One. 2021 Apr 7;16(4):e0249837. doi: 10.1371/journal.pone.0249837 (PMC8026056; doi:10.1371/journal.pone.0249837)
Supplement: S1 Table — (DOCX) [file pone.0249837.s001.docx]

| **Variables** | **Category** | **total** | **WCHS** | | **p-value** |  |
| --- | --- | --- | --- | --- | --- | --- |
|  |  |  | **< med** | **≥ med** | **X²** | **OR (95% CI)** |
| ***Baseline characteristics*** | | | | | | |
| Articles | N (%) | 104 (100.0) | 49 (100.0) | 55 (100.0) |  |  |
| WCHS | N, med (IQR) | 104, 10 (5.5-12) | 49, 5 (2-7) | 55, 12 (11-14) |  |  |
|  | < med # (%) | 49 (47.1) | 49 (100.0) | 0 (0.0) |  |  |
|  | ≥ med # (%) | 55 (52.9) | 0 (0.0) | 55 (100.0) |  |  |
| ***MRSA prevalence*** | | | | | | |
| MRSA prevalence | N, med (IQR) | 89, 0.33 (0.0727-0.436) | 39, 0.125 (0.06-0.436) | 50, 0.334 (0.13-0.44) | 0.3107^a^ |  |
|  | ≤ med # (%) | 44 (42.3) | 21 (42.9) | 23 (41.8) |  | reference (= 1) |
|  | > med # (%) | 45 (43.3) | 18 (36.7) | 27 (49.1) |  | 1.37 (0.591-3.172) |
|  | No data (%) | 15 (14.4) | 10 (20.4) | 5 (9.1) | 0.1997 | 0.457 (0.134-1.555) |
| ***MRSA Screening of patients*** | | | | | | |
| Patient screening | Not done (%) | 12 (11.5) | 4 (8.2) | 8 (14.5) | 0.0898 | reference (= 1) |
|  | Done (%) | 38 (36.5) | 14 (28.6) | 24 (43.6) |  | 0.857 (0.218-3.371) |
|  | No data (%) | 54 (51.9) | 31 (63.3) | 23 (41.8) |  | 0.371 (0.1-1.383) |
| ***Country*** |  |  |  |  |  |  |
| France | N (%) | 14 (13.5) | 4 (8.2) | 10 (18.2) | 0.1351 | 2.5 (0.73-8.561) |
| Japan | N (%) | 8 (7.7) | 8 (16.3) | 0 (0) | **0.002** | N/E |
| United Kingdom | N (%) | 15 (14.4) | 3 (6.1) | 12 (21.8) | **0.023** | 4.279 (1.13-16.207) |
| ***Type of hospital*** | | | | | | |
| Children´s hospital | N (%) | 6 (5.8) | 3 (6.1) | 3 (5.5) | 0.884 | 0.884 (0.17-4.599) |
| General hospital | N (%) | 30 (28.8) | 8 (16.3) | 22 (40) | **0.008** | 3.417 (1.348-8.66) |
| Teaching hospital | N (%) | 12 (11.5) | 5 (10.2) | 7 (12.7) | 0.6877 | 1.283 (0.379-4.34) |
| Long term care facility | N (%) | 5 (4.8) | 2 (4.1) | 3 (5.5) | 0.744 | 1.356 (0.217-8.47) |
| University hospital | N (%) | 37 (35.6) | 21 (42.9) | 16 (29.1) | 0.1433 | 0.547 (0.243-1.232) |
| ***Type of medical department*** | | | | | | |
| Neonatology | N (%) | 32 (30.8) | 12 (24.5) | 20 (36.4) | 0.1903 | 1.762 (0.752-4.13) |
| Surgery | N (%) | 27 (26) | 16 (32.7) | 11 (20) | 0.1418 | 0.516 (0.212-1.256) |
| Internal Medicine | N (%) | 10 (9.6) | 8 (16.3) | 2 (3.6) | **0.028** | 0.193 (0.039-0.96) |
| Gynecology | N (%) | 5 (4.8) | 2 (4.1) | 3 (5.5) | 0.744 | 1.356 (0.217-8.47) |
| Burn Unit | N (%) | 10 (9.6) | 1 (2) | 9 (16.4) | **0.013** | 9.391 (1.144-77.089) |
| Intensive Care Medicine | N (%) | 52 (50) | 26 (53.1) | 26 (47.3) | 0.5556 | 0.793 (0.367-1.715) |
| Long term care facility | N (%) | 11 (10.6) | 4 (8.2) | 7 (12.7) | 0.4500 | 1.641 (0.45-5.984) |
| Inpatient care | N (%) | 46 (44.2) | 24 (49) | 22 (40) | 0.3574 | 0.694 (0.319-1.511) |
| ***Source*** | | | | | | |
| Staff | N (%) | 11 (10.6) | 3 (6.1) | 8 (14.5) | 0.1633 | 0.383 (0.096-1.535) |
| Patients | N (%) | 23 (22.1) | 8 (16.3) | 15 (27.3) | 0.1794 | 1.922 (0.734-5.031) |
| No data mentioned | N (%) | 61 (58.7) | 35 (71.4) | 26 (47.3) | **0.013** | 0.359 (0.159-0.81) |
| Source remained unknown | N (%) | 10 (9.6) | 3 (6.1) | 7 (12.7) | 0.254 | 2.235 (0.545-9.17) |
| No data or source unknown | N (%) | 71 (68.3) | 38 (77.6) | 33 (60) | 0.0549 | 0.434 (0.184-1.027) |
| ***Route of transmission*** | | | | | | |
| Via staff | N (%) | 19 (18.3) | 5 (10.2) | 14 (25.5) | **0.045** | 3.005 (0.994-9.083) |
| Via patient | N (%) | 5 (4.8) | 3 (6.1) | 2 (3.6) | 0.554 | 0.579 (0.093-3.615) |
| No data mentioned | N (%) | 64 (61.5) | 36 (73.5) | 28 (50.9) | **0.018** | 0.375 (0.164-0.855) |
| Route remained unknown | N (%) | 12 (11.5) | 5 (10.2) | 7 (12.7) | 0.6877 | 1.283 (0.379-4.34) |
| No data or route unknown | N (%) | 76 (73.1) | 41 (83.7) | 35 (63.6) | **0.021** | 0.341 (0.134-0.87) |
| ***Outbreak characteristics*** | | | | | | |
| Duration of the outbreak [months] | N, med (IQR) | 94, 7 (2-14) | 42, 7 (2-13) | 52, 7 (3-16.5) | 0.6585^a^ |  |
|  | ≤ med # (%) | 50 (48.1) | 22 (44.9) | 28 (50.9) | 0.3095 | reference (= 1) |
|  | > med # (%) | 44 (42.3) | 20 (40.8) | 24 (43.6) |  | 0.943 (0.417-2.13) |
|  | No data (%) | 10 (9.6) | 7 (14.3) | 3 (5.5) |  | 0.337 (0.078-1.455) |
| Patients deceased while MRSA positive | N, med (IQR) | 56, 0(0-1) | 22, 0(0-1) | 34, 0(0-1) | 0.7727^a^ |  |
|  | ≤ med # (%) | 37 (35.6) | 15 (30.6) | 22 (40) | 0.2172 | reference (= 1) |
|  | > med # (%) | 19 (18.3) | 7 (14.3) | 12 (21.8) |  | 1.169 (0.374-3.655) |
|  | No data (%) | 48 (46.2) | 27 (55.1) | 21 (38.2) |  | 0.53 (0.222-1.265) |
| Patients affected by the outbreak | N, med (IQR) | 101, 12 (7-27) | 47, 11 (6-29) | 54, 12 (7-21) | 0.9538^a^ |  |
|  | ≤ med # (%) | 54 (51.9) | 24 (49) | 30 (54.5) | 0.713 | reference (= 1) |
|  | > med # (%) | 47 (45.2) | 23 (46.9) | 24 (43.6) |  | 0.835 (0.381-1.829) |
|  | No data (%) | 3 (2.9) | 2 (4.1) | 1 (1.8) |  | 0.4 (0.034-4.681) |
| Staff affected by the outbreak | N, med (IQR) | 53, 3 (1-6) | 16, 4.5 (1-5.5) | 37, 3 (1-6) | 0.7437^a^ |  |
|  | ≤ med # (%) | 24 (23.1) | 6 (12.2) | 18 (32.7) | **0.002** | reference (= 1) |
|  | > med # (%) | 29 (27.9) | 10 (20.4) | 19 (34.5) |  | 0.633 (0.191-2.103) |
|  | No data (%) | 51 (49) | 33 (67.3) | 18 (32.7) |  | 0.182 (0.061-0.54) |
| Individuals involved (total) | N, med (IQR) | 101, 15 (8-31) | 47, 14 (8-29) | 54, 16 (8-35) | 0.6189^a^ |  |
|  | ≤ med # (%) | 50 (48.1) | 24 (49) | 26 (47.3) | 0.756 | reference (= 1) |
|  | > med # (%) | 51 (49) | 23 (46.9) | 28 (50.9) |  | 1.124 (0.514-2.457) |
|  | No data (%) | 3 (2.9) | 2 (4.1) | 1 (1.8) |  | 0.462 (0.039-5.423) |
| Individuals infected (total) | N, med (IQR) | 67, 5 (3-11) | 28, 5.5 (3-12) | 39, 5 (2-11) | 0.8935^a^ |  |
|  | ≤ med # (%) | 34 (32.7) | 14 (28.6) | 20 (36.4) | 0.3408 | reference (= 1) |
|  | > med # (%) | 33 (31.7) | 14 (28.6) | 19 (34.5) |  | 0.95 (0.36-2.509) |
|  | No data (%) | 37 (35.6) | 21 (42.9) | 16 (29.1) |  | 0.533 (0.208-1.37) |

^a^ Wilcoxon rank sum test; OR, odds ratio – calculated by logistic regression; CI95, 95% confidence interval; N, number of outbreak reports; med, Median; IQR, interquartile range; N/A, not available; N/E, not estimable; WCHS, Weighted cumulative hygiene score
