# Supplementary material for: Infection control measures in nosocomial MRSA outbreaks—Results of a systematic analysis
Source: PLoS One. 2021 Apr 7;16(4):e0249837. doi: 10.1371/journal.pone.0249837 (PMC8026056; doi:10.1371/journal.pone.0249837)
Supplement: S2 Table — (DOCX) [file pone.0249837.s002.docx]

**S2 Table. Multivariate analysis by linear regression analysis for the weighted cumulative hygiene score.**

| **Factor** | **Regression**  **coefficient** | **Confidence interval (CI 95%)** | **p-value** |
| --- | --- | --- | --- |
| **Model including type of country** | | | |
| Constant term* | 12.96 | (10.64; 15.29) | 0.000 |
| Neonatology vs. other departments | 1.87 | (0.43; 3.32) | 0.011 |
| Burn unit vs. other departments | 3.46 | (1.13; 5.79) | 0.004 |
| Report from Japan vs. other countries | -3.85 | (-6.35; -1.36) | 0.002 |
| Report from UK vs. other countries | 2.70 | (0.79; 4.61) | 0.006 |
| No data on screening vs. no screening | -1.61 | (-3.77; 0.54) | 0.142 |
| Screening on admission vs. no screening | 0.45 | (-1.79; 2.68) | 0.696 |
| No. of personal ≤median | 4.02 | (2.32; 5.73) | 0.000 |
| No. of personal >median | 2.94 | (1.37; 4.51) | 0.000 |
| Data on staff not mentioned or unknown | 1 = reference |  |  |
| Source not mentioned vs. source mentioned | -3.14 | (-4.51; -1.78) | 0.000 |
| **Model not including type of country** | | | |
| Constant term* | 9.80 | (7.16; 12.45) | <0.001 |
| Neonatology vs. other departments | 1.56 | (0.01; 3.12) | 0.048 |
| Burn unit vs. other departments | 4.09 | (1.63; 6.54) | 0.001 |
| No data on screening vs. no screening | -2.65 | (-4.94; -0.35) | 0.024 |
| Screening on admission vs. no screening | 0.19 | (-2.23; 2.6) | 0.880 |
| No. of personal ≤median | 3.90 | (2.09; 5.71) | <0.001 |
| No. of personal >median | 3.35 | (1.68; 5.02) | <0.001 |
| Data on staff not mentioned or unknown | 1 = reference |  |  |
| Source not mentioned vs. source mentioned | -2.98 | (-4.5; -1.45) | <0.001 |

*this is the value for the hygiene-score calculated by the linear regression model without the presence of any factor considered in the model; ref, reference
