# Supplementary material for: Infection control measures in nosocomial MRSA outbreaks—Results of a systematic analysis
Source: PLoS One. 2021 Apr 7;16(4):e0249837. doi: 10.1371/journal.pone.0249837 (PMC8026056; doi:10.1371/journal.pone.0249837)
Supplement: S1 Appendix — (DOCX) [file pone.0249837.s003.docx]

**S1 Appendix. List of all MRSA outbreak reports included.**

1. Andersen BM, Lindemann R, Bergh K, Nesheim B-, Syversen G, Solheim N, et al. Spread of methicillin-resistant Staphylococcus aureus in a neonatal intensive unit associated with understaffing, overcrowding and mixing of patients. *Journal of Hospital Infection* 2002;**50**:18-24.

2. Andersen LP, Nielsen X. Methicillin-resistant Staphylococcus aureus transmission: unrecognised patient MRSA carriage. *Danish medical journal* 2015;**62**:A5047.

3. Baier C, Ipaktchi R, Ebadi E, Limbourg A, Mett TR, Vogt PM, et al. A multimodal infection control concept in a burn intensive care unit - lessons learnt from a meticillin-resistant Staphylococcus aureus outbreak. *J Hosp Infect* 2018;**98**:127-33.

4. Baldan R, Cavallerio P, Parlato C, Rocchetti A, Lomolino G, Vellini S, et al. Meticillin-resistant Staphylococcus aureus SCCmec type IV: nosocomial transmission and colonisation of healthcare workers in a neonatal intensive care unit. *J Hosp Infect* 2008;**69**:304-6.

5. Balslev U, Bremmelgaard A, Svejgaard E, Havstreym J, Westh H. An Outbreak of Borderline Oxacillin-Resistant Staphylococcus aureus (BORSA) in a Dermatological Unit. *Microbial drug resistance (Larchmont, N.Y.)* 2005;**11**:78.

6. Barbut F, Yezli S, Mimoun M, Pham J, Chaouat M, Otter JA. Reducing the spread of Acinetobacter baumannii and methicillin-resistant Staphylococcus aureus on a burns unit through the intervention of an infection control bundle. *Burns* 2012;**39**:395-403.

7. Berthelot P, Grattard F, Fascia P, Fichtner C, Moulin M, Lavocat MP, et al. Implication of a healthcare worker with chronic skin disease in the transmission of an epidemic strain of methicillin-resistant Staphylococcus aureus in a pediatric intensive care unit. *Infect Control Hosp Epidemiol* 2003;**24**:299-300.

8. Bertin ML, Vinski J, Schmitt S, Sabella C, Danziger-Isakov L, McHugh M, et al. Outbreak of methicillin-resistant Staphylococcus aureus colonization and infection in a neonatal intensive care unit epidemiologically linked to a healthcare worker with chronic otitis. *Infect Control Hosp Epidemiol* 2006;**27**:581-5.

9. Björholt I, Haglind E. Cost-savings achieved by eradication of epidemic methicillin-resistant Staphylococcus aureus (EMRSA)-16 from a large teaching hospital. *Eur J Clin Microbiol Infect Dis* 2004;**23**:688-95.

10. Boers SA, van Ess I, Euser SM, Jansen R, Tempelman FRH, Diederen BMW. An outbreak of a Multiresistant Methicillin-Susceptible Staphylococcus aureus (MR-MSSA) strain in a Burn Centre: The importance of routine molecular typing. *Burns* 2011;**37**:808-13.

11. Bratu S, Eramo A, Kopec R, Coughlin E, Ghitan M, Yost R, et al. Community-associated methicillin-resistant Staphylococcus aureus in hospital nursery and maternity units. *Emerging infectious diseases* 2005;**11**:808-13.

12. Carrier M, Marchand R, Auger P, Hébert Y, Pellerin M, Perrault LP, et al. Methicillin-resistant Staphylococcus aureus infection in a cardiac surgical unit. *J Thorac Cardiovasc Surg* 2002;**123**:40-4.

13. Cassone M, Campanile F, Pantosti A, Venditti M, Stefani S. Identification of a Variant "Rome Clone" of Methicillin-Resistant Staphylococcus aureus with Decreased Susceptibility to Vancomycin, Responsible for an Outbreak in an Intensive Care Unit. *Microbial drug resistance (Larchmont, N.Y.)* 2004;**10**:43.

14. Centers for Disease Control and Prevention. Community-associated methicillin-resistant Staphylococcus aureus infection among healthy newborns-Chicago and Los Angeles County, 2004. *MMWR Morb Mortal Wkly Rep* 2006;**55**:329-32.

15. Cetinkaya Y, Kocagöz S, Hayran M, Uzun O, Akova M, Gürsu G, et al. Analysis of a mini-outbreak of methicillin-resistant Staphylococcus aureus in a surgical ward by using arbitrarily primed-polymerase chain reaction. *J Chemother* 2000;**12**:138-44.

16. Christensen A, Scheel O, Urwitz K. Outbreak of Methicillin-resistant Staphylococcus aureus in a Norwegian Hospital. *Scandinavian Journal of Infectious Diseases* 2001;**33**:663-6.

17. Cimolai N. Ocular methicillin-resistant Staphylococcus aureus infections in a newborn intensive care cohort. *Am J Ophthalmol* 2006;**142**:183-4.

18. David MD, Kearns AM, Gossain S, Ganner M, Holmes A. Community-associated meticillin-resistant Staphylococcus aureus: nosocomial transmission in a neonatal unit. *Journal of Hospital Infection* 2006;**64**:244-50.

19. De Lassence A, Hidri N, ois Timsit Jean-Franc. Control and Outcome of a Large Outbreak of Colonization and Infection with Glycopeptide- Intermediate Staphylococcus aureus in an Intensive Care Unit. 2006;.

20. Drapeau CMJ, Angeletti C, Festa A, Petrosillo N. Role of previous hospitalization in clinically-significant MRSA infection among HIV-infected inpatients: results of a case-control study. *BMC Infect Dis* 2007;**7**:36.

21. Dryden M, Parnaby R, Dailly S, Lewis T, Davis-Blues K, Otter JA, et al. Hydrogen peroxide vapour decontamination in the control of a polyclonal meticillin-resistant Staphylococcus aureus outbreak on a surgical ward. *J Hosp Infect* 2008;**68**:190-2.

22. Dziekan G, Hahn A, Thüne K, Schwarzer G, Schäfer K, Daschner FD, et al. Methicillin-resistant Staphylococcus aureus in a teaching hospital: investigation of nosocomial transmission using a matched case-control study. *Journal of Hospital Infection* 2000;**46**:263-70.

23. Eckhardt C, Halvosa JS, Ray SM, Blumberg HM. Transmission of methicillin-resistant Staphylococcus aureus in the neonatal intensive care unit from a patient with community-acquired disease. *Infect Control Hosp Epidemiol* 2003;**24**:460-1.

24. Edgeworth JD, Yadegarfar G, Pathak S, Batra R, Cockfield JD, Wyncoll D, et al. An outbreak in an intensive care unit of a strain of methicillin-resistant Staphylococcus aureus sequence type 239 associated with an increased rate of vascular access device-related bacteremia. *Clin Infect Dis* 2007;**44**:493-501.

25. Embil JM, McLeod JA, Al-Barrak AM, Thompson GM, Aoki FY, Witwicki EJ, et al. An outbreak of methicillin resistant Staphylococcus aureus on a burn unit: potential role of contaminated hydrotherapy equipment. *Burns* 2001;**27**:681-8.

26. Faibis F, Laporte C, Fiacre A, Delisse C, Lina G, Demachy M, et al. An outbreak of methicillin-resistant Staphylococcus aureus surgical-site infections initiated by a healthcare worker with chronic sinusitis. *Infect Control Hosp Epidemiol* 2005;**26**:213-5.

27. Fascia P, Martin I, Mallaval F, Grattard F, Pozzetto B, Lucht F, et al. Possible implication of student nurses in the transmission of methicillin-resistant Staphylococcus aureus during a nosocomial outbreak. *Pathologie-biologie* 2003;**51**:479.

28. Fuchs PC, Kopp J, Häfner H, Kleiner U, Pallua N. MRSA—retrospective analysis of an outbreak in the burn centre Aachen. *Burns* 2002;**28**:575-8.

29. Garvey MI, Pichon B, Bradley CW, Moiemen NS, Oppenheim B, Kearns AM. Improved understanding of an outbreak of meticillin-resistant Staphylococcus aureus in a regional burns centre via whole-genome sequencing. *J Hosp Infect* 2016;**94**:401-4.

30. Garvey MI, Bradley CW, Holden KL, Oppenheim B. Outbreak of clonal complex 22 Panton–Valentine leucocidin-positive methicillin-resistant Staphylococcus aureus. *Journal of Infection Prevention* 2017;**18**:224-30.

31. Ghanem G, MD, Hachem RY, MD, Chemaly RF, MD, Dvorak T, BS, Hulten K, MD, Graviss, Linda, CIC, MT, et al. The role of molecular methods in the prevention of nosocomial methicillin-resistant Staphylococcus aureus clusters in cancer patients. *AJIC: American Journal of Infection Control* 2008;**36**:656-60.

32. Gould IM, Girvan EK, Browning RA, MacKenzie FM, Edwards GFS. Report of a hospital neonatal unit outbreak of community-associated methicillin-resistant Staphylococcus aureus. *Epidemiol Infect* 2009;**137**:1242-8.

33. Guerin F, Buu-Hoï A. Outbreak of Methicillin-Resistant Staphylococcus aureus with Reduced Susceptibility to Glycopeptides in a Parisian Hospital. *Journal of Clinical Microbiology* 2000;**38**:2985-8.

34. Harbarth S, Martin Y, Rohner P, Henry N, Auckenthaler R, Pittet D. Effect of delayed infection control measures on a hospital outbreak of methicillin-resistant Staphylococcus aureus. *J Hosp Infect* 2000;**46**:43-9.

35. Harberg D. Society for Healthcare Epidemiology of America guideline approach works to control a methicillin-resistant Staphylococcus aureus outbreak. *Infect Control Hosp Epidemiol* 2005;**26**:115-6.

36. Heym B, Le Moal M, Armand-Lefevre L, Nicolas-Chanoine M. Multilocus sequence typing (MLST) shows that the 'Iberian' clone of methicillin-resistant Staphylococcus aureus has spread to France and acquired reduced susceptibility to teicoplanin. *The Journal of antimicrobial chemotherapy* 2002;**50**:323.

37. Hitomi S, Kubota M, Mori N, Baba S, Yano H, Okuzumi K, et al. Control of a methicillin-resistant Staphylococcus aureus outbreak in a neonatal intensive care unit by unselective use of nasal mupirocin ointment. *Journal of Hospital Infection* 2000;**46**:123-9.

38. Iacobelli S, Colomb B, Bonsante F, Astruc K, Ferdynus C, Bouthet M, et al. Successful control of a Methicillin-resistant Staphylococcus aureus outbreak in a neonatal intensive care unit: a retrospective, before-after study. *BMC infectious diseases* 2013;**13**:440.

39. Imataki O, Makimoto A, Kato S, Bannai T, Numa N, Nukui Y, et al. Coincidental outbreak of methicillin‐resistant Staphylococcus aureus in a hematopoietic stem cell transplantation unit. *American Journal of Hematology* 2006;**81**:664-9.

40. James L, Gorwitz RJ, Jones RC, Watson JT, Hageman JC, Jernigan DB, et al. Methicillin-resistant Staphylococcus aureus infections among healthy full-term newborns. *Arch Dis Child Fetal Neonatal Ed* 2008;**93**:40.

41. Kanerva M, Blom M, Tuominen U, Kolho E, Anttila V-, Vaara M, et al. Costs of an outbreak of meticillin-resistant Staphylococcus aureus. *Journal of Hospital Infection* 2007;**66**:22-8.

42. Karchmer TB, Durbin LJ, Simonton BM, Farr BM. Cost-effectiveness of active surveillance cultures and contact/droplet precautions for control of methicillin-resistant Staphylococcus aureus. *J Hosp Infect* 2002;**51**:126-32.

43. Kaïret K, Ho E, Van Kerkhoven D, Boes J, Van Calenbergh S, Pattyn L, et al. USA300, A strain of community-associated methicillin-resistant Staphylococcus aureus, crossing Belgium's borders: outbreak of skin and soft tissue infections in a hospital in Belgium. *Eur J Clin Microbiol Infect Dis* 2017;**36**:905-9.

44. Kerttula A, Lyytikäinen O, Vuopio-Varkila J, Ibrahem S, Agthe N, Broas M, et al. Molecular Epidemiology of an Outbreak Caused by Methicillin-Resistant Staphylococcus aureus in a Health Care Ward and Associated Nursing Home. *J Clin Microbiol* 2005;**43**:6161-3.

45. Khoury J, Jones M, Grim A, Dunne WM, Fraser V. Eradication of methicillin-resistant Staphylococcus aureus from a neonatal intensive care unit by active surveillance and aggressive infection control measures. *Infect Control Hosp Epidemiol* 2005;**26**:616-21.

46. Kobayashi T, Nakaminami H, Ohtani H, Yamada K, Nasu Y, Takadama S, et al. An outbreak of severe infectious diseases caused by methicillin-resistant Staphylococcus aureus USA300 clone among hospitalized patients and nursing staff in a tertiary care university hospital. *J Infect Chemother* 2020;**26**:76-81.

47. Kossow A, Kampmeier S, Schaumburg F, Knaack D, Moellers M, Mellmann A. Whole genome sequencing reveals a prolonged and spatially spread nosocomial outbreak of Panton-Valentine leucocidin-positive meticillin-resistant Staphylococcus aureus (USA300). *J Hosp Infect* 2019;**101**:327-32.

48. Kotilainen P, Routamaa M, Peltonen R, Evesti P, Eerola E, Salmenlinna S, et al. Eradication of Methicillin-Resistant Staphylococcus aureus From a Health Center Ward and Associated Nursing Home. *Archives of Internal Medicine* 2001;**161**:859-63.

49. Kurlenda J, Grinholc M, Krzysztoń-Russjan J, Wiśniewska K. Epidemiological investigation of nosocomial outbreak of staphylococcal skin diseases in neonatal ward. *Antonie van Leeuwenhoek* 2009;**95**:387-94.

50. Lamanna O, Bongiorno D, Bertoncello L, Grandesso S, Mazzucato S, Pozzan GB, et al. Rapid containment of nosocomial transmission of a rare community-acquired methicillin-resistant Staphylococcus aureus (CA-MRSA) clone, responsible for the Staphylococcal Scalded Skin Syndrome (SSSS). *Italian journal of pediatrics* 2017;**43**:5.

51. Larssen KW, Jacobsen T, Bergh K, Tvete P, Kvello E, Scheel O. Outbreak of methicillin-resistant Staphylococcus aureus in two nursing homes in Central Norway. *Journal of Hospital Infection* 2005;**60**:312-6.

52. Le Coq M, Simon I, Sire C, Tissot-Guerraz F, Fournier L, Aho S, et al. [Epidemic of Staphylococcus aureus nosocomial infections resistant to methicillin in a maternity ward]. *Pathol Biol* 2001;**49**:16-22.

53. Lee TC, Moore C, Raboud JM, Muller MP, Green K, Tong A, et al. Impact of a mandatory infection control education program on nosocomial acquisition of methicillin-resistant Staphylococcus aureus. *Infect Control Hosp Epidemiol* 2009;**30**:249-56.

54. Lepelletier D, Lucet J-. Controlling meticillin-susceptible Staphylococcus aureus : not simply meticillin-resistant S. aureus revisited. *Journal of Hospital Infection* 2013;**84**:13-21.

55. Leroyer C, PharmD, Lehours P, PhD, Tristan A, PhD, Boyer F, PharmD, Marie V, PharmD, Elleau C, MD, et al. Outbreak in newborns of methicillin-resistant Staphylococcus aureus related to the sequence type 5 Geraldine clone. *AJIC: American Journal of Infection Control* 2016;**44**:e9-e11.

56. Mallaval F-, Carricajo A, Delavenna F, Recule C, Fonsale N, Manquat G, et al. Detection of an outbreak of methicillin-resistant Staphylococcus aureus with reduced susceptibility to glycopeptides in a French hospital. *Clinical Microbiology and Infection* 2004;**10**:459-61.

57. Manara S, Pasolli E, Dolce D, Ravenni N, Campana S, Armanini F, et al. Whole-genome epidemiology, characterisation, and phylogenetic reconstruction of Staphylococcus aureus strains in a paediatric hospital. *Genome medicine* 2018;**10**:82.

58. Maraha B, Halteren Jv, Verzijl JM, Wintermans RGF, Buiting AGM. Decolonization of methicillin-resistant Staphylococcus aureus using oral vancomycin and topical mupirocin. *Clinical Microbiology and Infection* 2002;**8**:671-5.

59. McAdams RM, Ellis MW, Trevino S, Rajnik M. Spread of methicillin‐resistant Staphylococcus aureus USA300 in a neonatal intensive care unit. *Pediatrics International* 2008;**50**:810-5.

60. McDonald J, Carriker C, Pien B, Trinh J, Engemann J, Harrell L, et al. Methicillin-Resistant Staphylococcus aureus Outbreak in an Intensive Care Nursery: Potential for Interinstitutional Spread. *The Pediatric Infectious Disease Journal* 2007;**26**:678-83.

61. Mine Y, Higuchi W, Taira K. Nosocomial outbreak of multidrug‐resistant USA300 methicillin‐resistant Staphylococcus aureus causing severe furuncles and carbuncles in Japan. *The Journal of Dermatology* 2011;**38**:1167-71.

62. Mitani N, Koizumi A, Sano R, Masutani T, Murakawa K, Mikasa K, et al. Molecular typing of methicillin-resistant Staphylococcus aureus by PCR-RFLP and its usefulness in an epidemiological study of an outbreak. *Japanese journal of infectious diseases* 2005;**58**:250.

63. Molina-Cabrillana J, del Rosario-Quintana C, Tosco-Núñez T, Dorta-Hung E, Quori A, Martín-Sánchez AM. Staphylococcus aureus resistente a la meticilina y a descolonizadores habituales con reservorio en un trabajador sanitario en un hospital de tercer nivel. *Enfermedades Infecciosas y Microbiología Clínica* 2012;**31**:511-5.

64. Morel A, Wu F, Della-Latta P, Cronquist A, Rubenstein D, Saiman L. Nosocomial transmission of methicillin-resistant Staphylococcus aureus from a mother to her preterm quadruplet infants. *Am J Infect Control* 2002;**30**:170-3.

65. Méan M, Mallaret MR, Andrini P, Recule C, Debillon T, Pavese P, et al. A neonatal specialist with recurrent methicillin-resistant Staphylococcus aureus (MRSA) carriage implicated in the transmission of MRSA to newborns. *Infect Control Hosp Epidemiol* 2007;**28**:625-8.

66. Nagao M, Iinuma Y, Suzuki M, Matsushima A, Takakura S, Ito Y, et al. First outbreak of methicillin-resistant Staphylococcus aureus USA300 harboring the Panton-Valentine leukocidin genes among Japanese health care workers and hospitalized patients. *American journal of infection control* 2010;**38**:e37-9.

67. Nakano M, Miyazawa H, Kawano Y, Kawagishi M, Torii K, Hasegawa T, et al. An Outbreak of Neonatal Toxic Shock Syndrome‐Like Exanthematous Disease (NTED) Caused by Methicillin‐Resistant Staphylococcus aureus (MRSA) in a Neonatal Intensive Care Unit. *Microbiology and Immunology* 2002;**46**:277-84.

68. Nambiar S, Herwaldt LA, Singh N. Outbreak of invasive disease caused by methicillin-resistant Staphylococcus aureus in neonates and prevalence in the neonatal intensive care unit. *Pediatric critical care medicine : a journal of the Society of Critical Care Medicine and the World Federation of Pediatric Intensive and Critical Care Societies* 2003;**4**:220-6.

69. Nguyen DM, Bancroft E, Mascola L, Guevara R, Yasuda L. Risk factors for neonatal methicillin-resistant Staphylococcus aureus infection in a well-infant nursery. *Infect Control Hosp Epidemiol* 2007;**28**:406-11.

70. Obaida A. Preventing Methicillin-resistant Staphylococcus aureus (MRSA) Transmission—A Private Room Helps but Is Not the Solution. *American Journal of Infection Control* 2010;**38**:e90-1.

71. Orendi JM, Coetzee N, Ellington MJ, Boakes E, Cookson BD, Hardy KJ, et al. Community and nosocomial transmission of Panton–Valentine leucocidin-positive community-associated meticillin-resistant Staphylococcus aureus : implications for healthcare. *Journal of Hospital Infection* 2010;**75**:258-64.

72. Otter JA, Klein JL, Watts TL, Kearns AM, French GL. Identification and control of an outbreak of ciprofloxacin-susceptible EMRSA-15 on a neonatal unit. *Journal of Hospital Infection* 2007;**67**:232-9.

73. Papastergiou P, Tsiouli E. Healthcare-associated transmission of Panton-Valentine leucocidin positive methicillin-resistant Staphylococcus aureus: the value of screening asymptomatic healthcare workers. *BMC infectious diseases* 2018;**18**:484.

74. Patel M, Thomas HC, Room J, Wilson Y, Kearns A, Gray J. Successful control of nosocomial transmission of the USA300 clone of community-acquired meticillin-resistant Staphylococcus aureus in a UK paediatric burns centre. *Journal of Hospital Infection* 2013;**84**:319-22.

75. Pina P, Marliere C, Vandenesch F, Bedos JP, Etienne J, Allouch PY. An outbreak of Staphylococcus aureus strains with reduced susceptibility to glycopeptides in a French general hospital. *Clin Infect Dis* 2000;**31**:1306-8.

76. Rampling A, Wiseman S, Davis L, Hyett AP, Walbridge AN, Payne GC, et al. Evidence that hospital hygiene is important in the control of methicillin-resistant Staphylococcus aureus. *Journal of Hospital Infection* 2001;**49**:109-16.

77. Rao JG, Qamruddin AO, Hassan IA, Burnie JP, Ganner M. Cluster of clinical isolates of epidemic methicillin-resistant Staphylococcus aureus (EMRSA) with a negative deoxyribonuclease (DNase) test-implications for laboratory diagnosis and infection control. *J Hosp Infect* 2002;**51**:238-9.

78. Rashid A, Solomon LK, Lewis HG, Khan K. Outbreak of epidemic methicillin-resistant Staphylococcus aureus in a regional burns unit: Management and implications. *Burns* 2006;**32**:452-7.

79. Rubin IM, Hansen TA, Klingenberg AM, Petersen AM, Worning P, Westh H, et al. A Sporadic Four-Year Hospital Outbreak of a ST97-IVa MRSA With Half of the Patients First Identified in the Community. *Frontiers in microbiology* 2018;**9**:1494.

80. Safdar N, Marx J, Meyer NA, Maki DG. Effectiveness of preemptive barrier precautions in controlling nosocomial colonization and infection by methicillin-resistant Staphylococcus aureus in a burn unit. *AJIC: American Journal of Infection Control* 2006;**34**:476-83.

81. Saiman L, Cronquist A, Wu F, Zhou J, Rubenstein D, Eisner W, et al. An outbreak of methicillin-resistant Staphylococcus aureus in a neonatal intensive care unit. *Infect Control Hosp Epidemiol* 2003;**24**:317-21.

82. Saiman L, O'Keefe M, Graham PL. Hospital Transmission of Community-Acquired Methicillin-Resistant Staphylococcus aureus among Postpartum Women. *Clinical Infectious Diseases* 2003;**37**:1313-9.

83. Samuel R, Axelrod P, John KS, Fekete T, Alexander S, McCarthy J, et al. An outbreak of mediastinitis among heart transplant recipients apparently related to a change in the united network for organ sharing guidelines. *Infect Control Hosp Epidemiol* 2002;**23**:377-81.

84. Saunders A, Panaro L, McGeer A. A Nosocomial Outbreak of Community-Associated Methicillin-Resistant Staphylococcus aureus among Healthy Newborns and Postpartum Mothers. *The Canadian journal of infectious diseases & medical microbiology = Journal canadien des maladies infectieuses et de la microbiologie medicale* 2007;**18**:128-32.

85. Sax H, Posfay-Barbe K, Harbarth S, Francois P, Touveneau S, Pessoa-Silva CL, et al. Control of a cluster of community-associated, methicillin-resistant Staphylococcus aureus in neonatology. *Journal of Hospital Infection* 2006;**63**:93-100.

86. Schultsz C, Meester HHM, Kranenburg AMH, Savelkoul PHM, Boeijen-Donkers LEA, Kaiser AM, et al. Ultra-sonic nebulizers as a potential source of methicillin-resistant Staphylococcus aureus causing an outbreak in a university tertiary care hospital. *Journal of Hospital Infection* 2003;**55**:269-75.

87. Shaw BE, Boswell T, Byrne JL, Yates C, Russell NH. Clinical impact of MRSA in a stem cell transplant unit: analysis before, during and after an MRSA outbreak. *Bone Marrow Transplant* 2007;**39**:623-9.

88. Shiojima T, Ohki Y, Nako Y, Morikawa A, Okubo T, Iyobe S. Immediate control of a methicillin-resistant Staphylococcus aureus outbreak in a neonatal intensive care unit. *J Infect Chemother* 2003;**9**:243-7.

89. Silvestri L, Milanese M, Oblach L, Fontana F, Gregori D, Guerra R, et al. Enteral vancomycin to control methicillin-resistant Staphylococcus aureus outbreak in mechanically ventilated patients. *AJIC: American Journal of Infection Control* 2002;**30**:391-9.

90. Singh K, Gavin P, Vescio T. Microbiologic Surveillance Using Nasal Cultures Alone Is Sufficient for Detection of Methicillin-Resistant Staphylococcus aureus Isolates in Neonates. *Journal of Clinical Microbiology* 2003;**41**:2755-7.

91. Steensels D, Deplano A, Denis O, Simon A, Verroken A. MALDI-TOF MS typing of a nosocomial methicillin-resistant Staphylococcus aureus outbreak in a neonatal intensive care unit. *Acta Clin Belg* 2017;**72**:219-25.

92. Stock NK, Petráš P, Melter O, Kapounová G, Vopalková P, Kubele J, et al. Importance of Multifaceted Approaches in Infection Control: A Practical Experience from an Outbreak Investigation. *PloS one* 2016;**11**:e0157981.

93. Szczepanik A, Kozioł-Montewka M, Al-Doori Z, Morrison D, Kaczor D. Spread of a single multiresistant methicillin-resistant Staphylococcus aureus clone carrying a variant of staphylococcal cassette chromosome mec type III isolated in a university hospital. *Eur J Clin Microbiol Infect Dis* 2007;**26**:29-35.

94. Sánchez García M, De la Torre, María Angeles, Morales G, Peláez B, Tolón MJ, Domingo S, et al. Clinical outbreak of linezolid-resistant Staphylococcus aureus in an intensive care unit. *JAMA* 2010;**303**:2260-4.

95. Tadros MA, Williams VR, Plourde S, Callery S, Simor AE, Vearncombe M. Risk factors for Staphylococcus aureus surgical site infection during an outbreak in patients undergoing cardiovascular surgery. *Am J Infect Control* 2013;**41**:509-12.

96. Tansel O, Kuloglu F, Mutlu B, Anthony RM, Uyar A, Vahaboglu H, et al. A methicillin-resistant Staphylococcus aureus outbreak in a new University hospital due to a strain transferred with an infected patient from another city six months previously. *New Microbiol* 2003;**26**:175-80.

97. Teare L, Shelley OP, Millership S, Kearns A. Outbreak of Panton-Valentine leucocidin-positive meticillin-resistant Staphylococcus aureus in a regional burns unit. *J Hosp Infect* 2010;**76**:220-4.

98. Ugolotti E, Di Marco E, Bandettini R, Biassoni R. Genomic characterization of a paediatric MRSA outbreak by next-generation sequencing. *J Hosp Infect* 2018;**98**:155-60.

99. Vos M, Behrendt M, Melles D, Mollema F, Groot W, Parlevliet G, et al. 5 years of experience implementing a methicillin-resistant Staphylococcus aureus search and destroy policy at the largest university medical center in the Netherlands. *Infection Control & Hospital Epidemiology* 2009;**30**:977-84.

100. Wagenlehner FME, Naber KG, Bambl E, Raab U, Wagenlehner C, Kahlau D, et al. Management of a large healthcare-associated outbreak of Panton-Valentine leucocidin-positive meticillin-resistant Staphylococcus aureus in Germany. *J Hosp Infect* 2007;**67**:114-20.

101. Williams, Victoria R., BSc, BASc, CIC, Callery, Sandra, RN, MHSc, CIC, Vearncombe, Mary, MD, FRCPC, Simor, Andrew E., MD, FRCPC. The role of colonization pressure in nosocomial transmission of methicillin-resistant Staphylococcus aureus. *AJIC: American Journal of Infection Control* 2009;**37**:106-10.

102. Wulf MWH, Markestein A, Linden, F. T. van der, Voss A, Klaassen C, Verduin CM. First outbreak of methicillin-resistant Staphylococcus aureus ST398 in a Dutch hospital, June 2007. *Euro Surveillance : Bulletin Europeen sur les Maladies Transmissibles* 2008;**13**:8051.

103. Young LS, Perdreau-Remington F, Winston LG. Clinical, Epidemiologic, and Molecular Evaluation of a Clonal Outbreak of Methicillin-Resistant Staphylococcus aureus Infection. Clinical Infectious Diseases 2004;38:1075-83.
