# Supplementary material for: Infection control measures in nosocomial MRSA outbreaks—Results of a systematic analysis
Source: PLoS One. 2021 Apr 7;16(4):e0249837. doi: 10.1371/journal.pone.0249837 (PMC8026056; doi:10.1371/journal.pone.0249837)
Supplement: S3 Appendix — (DOC) [file pone.0249837.s005.doc]

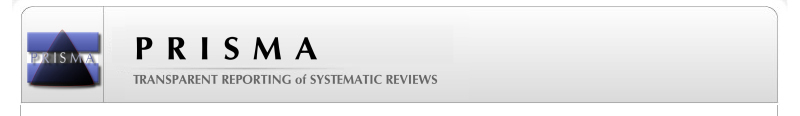
**PRISMA 2009 Flow Diagram**

**Screening**

**Included**

**Eligibility**

**Identification**

**Source A:**

Outbreak Database

Additional records identified through other sources
(n = 0)

- Checking for **duplicates**
- Screening of **titles**
- Screening of **abstracts**

Studies included in qualitative synthesis
(n = 104)

Studies included in quantitative synthesis (meta-analysis)
(n = 104)

**Source B:**

Pubmed

**Source C:**

Embase

**Source D:**

Reference lists

- Checking full text for solitaire **outbreak description**
- Checking for **inclusion criteria**
- **language** (English, French, or German)
- **country** (US, Canada, Europe, or Japan)
- **date** (published after the year 2000)

**Univariate** and **multivariate** analysis
